# Cessation of seizure-like oscillations by periodic stimulation in a neuron model with dynamic ion concentrations

**DOI:** 10.1186/1471-2202-13-S1-P182

**Published:** 2012-07-16

**Authors:** Jeremy Owen, John R Cressman, Ernest Barreto

**Affiliations:** 1 Cornell University, Ithaca, NY 14853, USA; 2 School of Physics, Astronomy, and Computational Sciences, and The Krasnow Institute for Advanced Study, George Mason University, Fairfax, VA 22030, USA

## 

**Figure 1:**
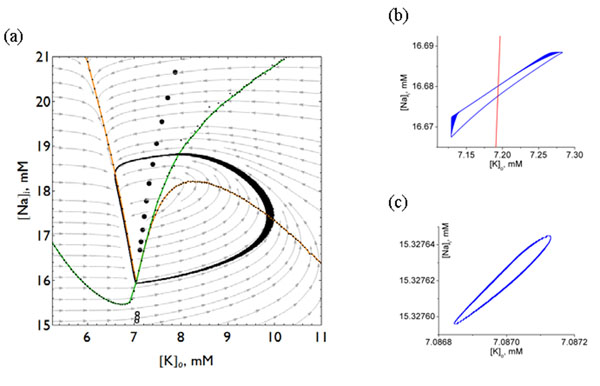
Under stimulation, the model trajectory approaches small loops at locations indicated in (a) by closed(excitatory) or open(inhibitory) symbols for various values of stimulation frequency. Frequency increases upwards (downwards) for excitatory (inhibitory) stimulations. (b and c) Close ups of the small trajectories produced by excitatory and inhibitory stimulation.

Here we describe the effect of adding periodic forcing to a simple model of a generic neuron with slow Na+ and K+ concentration dynamics [1]. With no stimulation, the model exhibits bursting due to the gradual modulation of ion concentrations, this limit cycle is shown in panel (a) of Figure 1. We have identified a range of parameter values where periodic stimulation can stop seizure-like bursting by freezing the slow ionic dynamics. Instead of following the large “bursting” limit cycle in the Na+/K+ phase plane, the stimulation forces the model into much smaller loops in the ion concentration space. These small trajectories are shown in panels (a) and (b) of Figure 1 for excitatory and inhibitory stimulation respectively.

In the case of excitatory stimulation, the model continues to exhibit spikes in voltage, but only at the stimulation frequency—which can be made much lower than the rate of spontaneous spiking seen in bursting. For other parameter values, the addition of stimulation can reduce the amplitude of the bursts, or in some cases, induce bursts. These results may give insight into previous studies which showed that stimulation via electrodes can stop seizures in slice preparations [2], could help explain the mechanism of action of vagus nerve stimulation as treatment for epilepsy [3], and may provide novel avenues for treatment of epilepsy.
